# Health-related quality of life in children with multiple endocrine neoplasia (MEN) and their siblings

**DOI:** 10.1210/clinem/dgag094

**Published:** 2026-03-04

**Authors:** Daniël Zwerus, Annemarie A Verrijn Stuart, Hanneke M van Santen, Gerlof D Valk, Christiaan de Bruin, Theo C J Sas, Gianni Bocca, Christiaan F Mooij, Hedi L Claahsen – van der Grinten, Saartje Straetemans, Sasja A Schepers, Rachel S van Leeuwaarde

**Affiliations:** Department of Endocrine Oncology, University Medical Center Utrecht, 3584 CX Utrecht, The Netherlands; Wilhelmina Children's Hospital, University Medical Center Utrecht, 3584 EA Utrecht, The Netherlands; Wilhelmina Children's Hospital, University Medical Center Utrecht, 3584 EA Utrecht, The Netherlands; Wilhelmina Children's Hospital, University Medical Center Utrecht, 3584 EA Utrecht, The Netherlands; Princess Máxima Center for Pediatric Oncology, 3584 CS Utrecht, The Netherlands; Department of Endocrine Oncology, University Medical Center Utrecht, 3584 CX Utrecht, The Netherlands; Willem-Alexander Children's Hospital, LUMC, 2333 ZC Leiden, The Netherlands; Department of Pediatric Endocrinology, Erasmus Medical Center—Sophia's Children's Hospital, 3015 AA Rotterdam, The Netherlands; Diabeter Center for Pediatric and Adult Diabetes Care and Research, 3011 TA Rotterdam, The Netherlands; Beatrix Children's Hospital, Department of Pediatrics, University Medical Center Groningen, University of Groningen, 9713 GZ Groningen, The Netherlands; Department of Pediatric Endocrinology, Emma's Children's Hospital, Amsterdam University Medical Center, University of Amsterdam and Vrije Universiteit, 1105 AZ Amsterdam, The Netherlands; Department of Pediatrics, Amalia's Children's Hospital, Radboud UMC, 6525 GA Nijmegen, The Netherlands; MosaKids Children's Hospital, Maastricht UMC, 6229 HX Maastricht, The Netherlands; Wilhelmina Children's Hospital, University Medical Center Utrecht, 3584 EA Utrecht, The Netherlands; Department of Endocrine Oncology, University Medical Center Utrecht, 3584 CX Utrecht, The Netherlands

**Keywords:** multiple endocrine neoplasia, psychosocial, quality of life, children, siblings, parents

## Abstract

**Context:**

Multiple Endocrine Neoplasia (MEN) syndromes are rare autosomal dominant hereditary tumor predisposition syndromes affecting multiple family members. Carriers undergo health surveillance from early childhood onwards. Carriership or surveillance may influence Health-Related Quality of Life (HRQoL) for patients or their families.

**Objective:**

To evaluate HRQoL in children and adolescents with genetically confirmed MEN1, MEN2A, and MEN2B, and to compare outcomes with their siblings without MEN and healthy Dutch norms. Secondary aims were to explore associations between HRQoL and clinical characteristics.

**Methods:**

This nationwide cross-sectional study included 77 children with MEN (5-18 years) and 26 siblings (8-29 years). The Pediatric Quality of Life Inventory (PedsQL) was used to assess HRQoL, with children completing self-report questionnaires and parents providing proxy reports for their children with MEN. Sociodemographic and clinical data were obtained from medical records.

**Results:**

Children with MEN1 and MEN2A reported HRQoL comparable to siblings and healthy Dutch norms. Only children with MEN2B showed significantly lower physical HRQoL. No gender or age effects were observed. Parent-proxy scores were significantly higher than child self-reports on all domains, except for emotional functioning. Children with MEN1 and MEN2B having clinical MEN-related manifestations had significantly lower physical, social, and school functioning scores than those without clinical manifestations.

**Conclusion:**

Overall, children with MEN reported HRQoL comparable to siblings and age-matched Dutch norms, except for reduced physical functioning in MEN2B. Clinical manifestations negatively affected physical, social, and school functioning, suggesting that children with the onset of disease may benefit from closer monitoring and targeted psychosocial support.

Multiple Endocrine Neoplasia Type 1 and 2 (MEN) are rare inherited tumor predisposition syndromes that predispose carriers to develop various endocrine and non-endocrine tumors (1). MEN type 1 (MEN1) is caused by pathogenic variants in the *MEN1* suppressor gene and MEN type 2 (MEN2A and MEN2B) by variants in the *RET* proto-oncogene (2, 3). Although classified together as MEN syndromes, they are genetically and clinically distinct entities. The main characteristics of the different subtypes of MEN are listed in Table 1. All types of MEN follow an autosomal dominant inheritance pattern, yet MEN2B often occurs de novo. Expression varies widely in age of onset, type of manifestations, and disease severity; depending on the subtype of MEN, partly reflecting known genotype-phenotype correlations across MEN subtypes (4-6). Medullary thyroid cancer (MTC) in MEN2A, and particularly in MEN2B, can manifest early, sometimes within the first years of life, warranting prophylactic thyroidectomy in infancy or early childhood (7).

**Table 1:** Multiple endocrine neoplasia (MEN) and its main manifestations and shared aspects (2, 3, 7, 34-36)

MEN1	MEN2A	MEN2B
Hyperparathyroidism Pituitary tumors Duodenopancreatic NETs Lung/thymic NETs Adrenal tumors	Medullary Thyroid Cancer Pheochromocytoma Hyperparathyroidism	Medullary thyroid cancer Pheochromocytoma Multiple neuromas Ganglioneuromatosis of the gastrointestinal tract Facial characteristics
**Family-wide impact**		
**Autosomal dominant nature**		
**Presence of a clinical screening protocol**		
**Varying penetrance from childhood to adulthood**		

Genetic counseling and testing are recommended for all first-degree relatives of mutation carriers, as they are at risk of carrying the same pathogenic variant (7, 8). Predictive testing in childhood enables early inclusion in screening programs, distinguishing MEN from other tumor predisposition syndromes that are usually recognized only after clinical manifestations occur (9). The screening protocols, tailored to the MEN subtype, are periodically updated based on emerging evidence and expert consensus, and consist of age-specific biochemical and imaging surveillance (7, 10). Despite limited evidence on the psychosocial impact of hereditary tumor predisposition in children, both psychological benefits and harms of tumor surveillance and genetic testing have been reported (11, 12). The aim of screening is early disease detection, when treatment is most effective. However, repeated screening may heighten uncertainty and cause anxiety (12-14). The combination of high disease penetrance, potential early onset of disease-related manifestations, and lifelong surveillance can impose a significant physical and psychological burden on both children and their families. It has been shown that adults with MEN1 have a reduced Health-Related Quality of Life (HRQoL), particularly those with high disease or treatment burden, and frequently had fear of recurrence (15-17).

Up to now, there is a lack of empirical research on the HRQoL of children with MEN syndrome (18). The relevance of research into the psychosocial well-being of children and adolescents with rare diseases has been increasingly emphasized in recent years (19). Understanding their HRQoL is essential, as these children grow up with a lifelong condition that may affect not only physical health but also emotional wellbeing, social development, and family dynamics (20). The objective of this study was to assess the HRQoL in children diagnosed with a MEN syndrome and to compare it to HRQoL as reported by their siblings without MEN and to normative population data. Second, we aimed to explore associations between HRQoL and clinical characteristics in the patient group. Mapping these outcomes will help to identify specific needs and inform psychosocial care, with the ultimate goal of improving patient-centered care and family wellbeing in this rare hereditary disease context.

## Methods

### Participants

In a nationwide Dutch study, all families known in one of the Dutch University Medical Centers, with one or more children aged 5 to 18 years with a genetically confirmed diagnosis of MEN1, MEN2A, or MEN2B were invited to participate in this study. Family members were eligible to participate, including parents or legal guardians, regardless of whether they themselves carried a MEN mutation, and siblings aged between 8 and 30 years without a MEN predisposition. This broader age range for siblings was chosen because, unlike some measures used for children with MEN, all sibling questionnaires were validated for young adults. Children aged 8 years and older, who were aware of their MEN diagnosis, completed the self-report questionnaires themselves, whereas for children aged 5 to 7 years, only parent proxy-reports were obtained. Participants were recruited between December 2023 and May 2025 across all seven Dutch University Medical Centers: Wilhelmina Children's Hospital, University Medical Center Utrecht; Sophia Children's Hospital, Erasmus Medical Center, Rotterdam; Beatrix Children's Hospital, University Medical Center Groningen; Emma Children's Hospital, Amsterdam University Medical Center; Willem-Alexander Children's Hospital, Leiden University Medical Center; Amalia Children's Hospital, Radboud University Medical Center, Nijmegen; and MosaKids Children's Hospital, Maastricht University Medical Center+. The study was approved by all local Medical Ethics Committees.

### Study design

**Figure 1:**
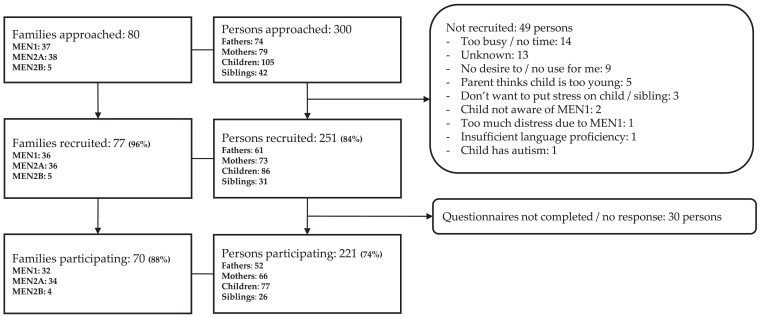
Flowchart of the study population.

A cross-sectional study was conducted in which affected families were invited to complete multiple online questionnaires addressing various psychosocial domains. In addition, clinical characteristics were collected, such as age, sex, level of education of the parents, MEN genotype, surgical history, onset of disease manifestations, mutation risk category, and family history of MEN-related deaths. Eligible families were initially approached by their primary treating physician at participating University Medical Centers and subsequently contacted by the coordinating researcher by phone to provide detailed study information. Following a 2-week reflection period, families were re-contacted for a follow-up call during which verbal informed consent was obtained and documented in accordance with ethical guidelines. Figure 1 provides an overview of participant inclusion. After consent, participants received personalized login codes via email to access a secure study website and to complete the questionnaires, confirming consent electronically before participation. To optimize response rates, reminder emails were sent after 2 and 4 weeks, and non-responders were followed up by telephone calls or reminded by their physician.

### Questionnaires

To assess HRQoL, the Dutch version of the Pediatric Quality of Life Inventory 4.0 (PedsQL) was used. The PedsQL is a standardized measure of HRQoL for children, adolescents, and young adults. It contains 23 items covering four domains: physical, emotional, social, and school functioning (21). The PedsQL distinguishes between age-specific versions corresponding to the age categories used in this study (5-18). By providing age-specific normative population data based on healthy reference samples, the PedsQL allows comparison of individual scores with established population norms (22). The PedsQL has demonstrated good internal consistency and construct validity across pediatric populations, with Cronbach's α values typically exceeding 0.70 for both self- and parent-proxy reports (21). The Dutch version of the PedsQL has demonstrated adequate psychometric properties comparable to the original version and has been widely used as a measure of HRQoL in pediatric research in the Netherlands (22). For each PedsQL scale, raw item scores were linearly transformed to a 0-100 scale, with higher scores indicating better HRQoL, following the scoring procedures outlined by Varni et al (21). Scale scores were calculated as the mean of transformed items within each subscale (Physical, Emotional, Social, and School Functioning), provided that at least 50% of items were completed. A Psychosocial Health Summary Score (mean of Emotional, Social, and School Functioning subscales) and a Total Scale Score (mean of all 23 items) were also derived. The scoring and interpretation procedures were identical for both self-report and parent-proxy versions. In addition to HRQoL assessment, parental educational level was classified into three categories: low (primary/lower secondary), intermediate (upper secondary/vocational), and high (higher professional/university).

### Statistical analysis

Descriptive statistics (Means, Medians, Standard Deviations, and percentages) were computed for all core domains (Physical, Emotional, Social, and School Functioning), and for the Psychosocial Health Summary Score and Total Scale Scores. In addition, exploratory univariate analyses were conducted to examine differences between groups; one-sample sign tests were used to compare HRQoL outcomes of affected children with age-matched Dutch healthy norms. Kruskal–Wallis *H* tests were applied to compare scores between MEN subtypes (MEN1, MEN2A, MEN2B) and different age categories (5-18). Pairwise comparisons using Dunn's procedure revealed the direction of the observed findings (23). Mann–Whitney *U* tests were performed to assess differences between children with a genetic predisposition and their unaffected siblings, and between boys and girls within these groups. Wilcoxon signed-rank tests were used to compare proxy- and self-reports. Finally, to further examine the group with the lowest HRQoL scores, participants within the lowest 20th percentile of HRQoL scores in each domain were classified as the *at-risk* group, and the remaining 80% as the *normal* group. Depending on a minimum expected cell count of five, either Chi-square or Fisher's exact tests were used to examine associations between the proportion of children with *at-risk* HRQoL scores and clinical characteristics. All statistical tests were conducted only after verifying that the underlying assumptions were met.

## Results

### Response rate

A total of 80 families (consisting of 300 family members; 74 fathers, 79 mothers, 105 affected children and 42 siblings) were approached. Of those, 70 families (consisting of 221 family members; 52 fathers, 66 mothers, 77 affected children and 26 siblings) completed the questionnaires (total response rate 74%; see Fig. 1).

### Sample characteristics

At inclusion, affected children (N = 77) were on average 12.5 years of age (SD = 3.5, range 5-18). All children either had a genetically confirmed MEN1, MEN2A or MEN2B mutation. Siblings without MEN (N = 26) were on average 15.8 years (SD = 4.9, range 8-29). MEN2A (N = 44) was the largest pediatric subgroup, whereas MEN2B (N = 4) was rare and characterized by markedly earlier onset of manifestations and first surgery. Risk classifications based on gene mutations were assigned according to the ATA risk stratification guidelines (24). Clinical manifestations corresponded to expected genotype profiles. Surgical rates were high in MEN2A and MEN2B but low in MEN1. MEN1-related procedures included parathyroidectomy, transsphenoidal pituitary resection, and distal pancreatectomy, whereas surgery in MEN2A and MEN2B consisted of (prophylactic) thyroidectomy. Detailed demographic and clinical characteristics are depicted in Table 2.

**Table 2:** Background characteristics

Characteristics	Total No. (%)	MEN1 No. (%)	MEN2A No. (%)	MEN2B No. (%)
*Pediatric patients*	77 (100)	29 (38)	44 (57)	4 (5)
**Gender**				
Female	35 (45)	11 (38)	21 (48)	3 (75)
Male	42 (55)	18 (62)	23 (52)	1 (25)
**Age at inclusion in years, mean (SD)**	12.47 (3.46)	13.45 (3.42)	11.73 (3.42)	13.50 (2.65)
**Age categories**				
5-7	9 (11)	2 (7)	7 (16)	—
8-12	32 (42)	10 (34)	20 (45)	2 (50)
13-18	36 (47)	17 (59)	17 (39)	2 (50)
**Onset manifestations**				
Yes	16 (21)	12 (41)	—	4 (100)
No	61 (79)	17 (59)	44 (100)	—
**Age first onset in years, mean (SD)**	12.53 (4.40)	13.60 (3.44)	—	4.75 (2.06)
**Clinical Manifestations**				
Hyperparathyroidism	—	8 (28)	—	—
Pancreas NET	—	4 (14)	—	—
Pituitary adenoma	—	5 (17)	—	—
MTC	—	—	—	1 (25)
Other*^a^*	—	—	—	3 (75)
ATA Risk Classification (24)				
Moderate	—	—	17 (39)	—
High (2A)/Highest (2B)	—	—	27 (61)	4 (100)
Surgery				
Yes	52 (68)	5 (17)	43 (98)	4 (100)
No	25 (32)	24 (83)	1 (2)	0 (0)
**Age first surgery in years, mean (SD)**	4.06 (4.22)	13.20 (4.15)	3.24 (2.85)	1.5 (3.00)
**Familial deaths due to MEN (≤ 2nd deg.)**				
Yes	18 (23)	12 (42)	6 (18)	—
No	56 (73)	14 (48)	38 (82)	4 (100)
Unknown	3 (4)	3 (10)	—	—
*Siblings*	26 (100)	13 (50)	11 (42)	2 (8)
**Age in years, mean (SD)**	15.77 (4.86)	16.77 (5.34)	14.91 (4.68)	14.00 (1.41)
Gender				
Female	13 (50)	9 (69)	4 (31)	—
Male	13 (50)	4 (31)	7 (54)	2 (15)
*Parents*	118 (100)	53 (45)	57 (48)	8 (7)
**Age in years, mean (SD)**	45.35 (6.70)	46.71 (7.33)	43.83 (5.92)	48.50 (5.75)
Gender				
Male (N with mutation)	52 (23)	23 (10)	25 (13)	4 (0)
Female (N with mutation)	66 (34)	30 (18)	32 (16)	4 (0)
**Affected**				
Yes	57 (48)	28 (53)	29 (51)	—
No	61 (52)	25 (47)	28 (49)	8 (100)
**Education level**				
Low	—	—	—	—
Intermediate	25 (21)	10 (18)	14 (25)	1 (12)
High	43 (36)	21 (40)	19 (33)	3 (38)
Missing*^b^*	50 (43)	22 (42)	24 (42)	4 (50)

^*a*^Gastrointestinal ganglioneuromatosis and multiple neuromas.

^*b*^Only one parent per family completed a questionnaire that included data on education level.

### Overall group comparisons to normative data

HRQoL outcomes across age categories and MEN subtypes are presented as medians and interquartile ranges (IQR) in Table 3, benchmarked against established healthy norms and siblings without MEN. Corresponding mean and standard deviation (SD) values are provided in Table S1 (25). Children and adolescents diagnosed with MEN syndrome reported comparable HRQoL scores to age-matched healthy norms from the Dutch population across all PedsQL domains (Table 3). There were no differences (*P* > .05) in median scores across all domains between the MEN group and the normative data for any of the age groups (5-7, 8-12, and 13-18 years).

**Table 3:** HRQoL outcomes medians and interquartile range of children with MEN, healthy peers and siblings without MEN

		Physical	Emotional	Social	School/Work	Psychosocial	Total
	N	Mdn (IQR)	Mdn (IQR)	Mdn (IQR)	Mdn (IQR)	Mdn (IQR)	Mdn (IQR)
**Total**	**77**	**94 (84-100)**	**85 (65-95)**	**95 (80-100)**	**80 (65-90)**	**83 (73-93)**	**88 (77-95)**
5-7	9	97 (88-100)	75 (65-98)	95 (83-100)	90 (85-100)	85 (80-97)	90 (84-98)
8-12	32	94 (85-100)	83 (61-90)	98 (80-100)	70 (61-90)	83 (68-93)	86 (75-94)
13-18	36	95 (82-100)	85 (70-95)	93 (80-100)	80 (65-90)	84 (72-92)	88 (76-95)
**MEN1**	**29**	**94 (88-100)**	**85 (73-95)**	**90 (78-100)**	**85 (65-93)**	**85 (75-93)**	**89 (77-95)**
5-7	2	91 (−)	70 (−)	83 (−)	88 (−)	80 (−)	84 (−)
8-12	10	100 (90-100)	85 (65-100)	100 (94-100)	85 (64-100)	89 (81-96)	91 (85-97)
13-18	17	94 (80-100)	80 (70-95)	90 (75-95)	80 (63-90)	85 (68-92)	89 (73-05)
**MEN2A**	**44**	**97 (84-100)**	**85 (65-95)**	**95 (81-100)**	**80 (65-90)**	**84 (72-93)**	**88 (77-95)**
5-7	7	100 (84-100)	85 (65-100)	100 (90-100)	100 (85-100)	95 (83-98)	91 (88-99)
8-12	20	94 (82-100)	78 (60-90)	95 (76-100)	70 (61-89)	81 (65-93)	85 (70-93)
13-18	17	97 (88-100)	85 (68-95)	95 (88-100)	80 (65-90)	85 (76-93)	88 (78-95)
**MEN2B**	**4**	**72 (66-88)**	**78 (64-91)**	**83 (76-96)**	**65 (53-78)**	**75 (71-82)**	**77 (71-79)**
8-12	2	84 (−)	70 (−)	90 (−)	65 (−)	75 (−)	78 (−)
13-18	2	66 (−)	85 (−)	80 (−)	65 (−)	77 (−)	73 (−)
**Healthy peers** (37, 38)							
5-7	274	94 (88-100)	80 (65-90)	95 (75-100)	90 (75-100)	87 (75-95)	88 (79-96)
8-12	475	97 (88-100)	80 (70-95)	90 (75-100)	85 (70-95)	83 (73-93)	87 (78-95)
13-17	491	97 (88-100)	85 (70-100)	90 (75-100)	80 (65-95)	83 (72-93)	87 (78-96)
18-25	385	94 (81-100)	75 (65-90)	90 (78-100)	85 (70-95)	83 (73-93)	87 (77-93)
**Siblings**	**26**	**97 (91-100)**	**90 (65-96)**	**95 (80-100)**	**99 (75-95)**	**89 (79-94)**	**92 (81-95)**
8-12	8	100 (95-100)	98 (83-100)	100 (100-100)	95 (91-100)	98 (93-100)	98 (94-100)
13-17	10	97 (93-100)	88 (76-91)	93 (79-100)	80 (73-88)	88 (74-92)	92 (80-93)
18-30	8	92 (80-99)	65 (61-95)	93 (81-99)	88 (75-94)	83 (75-90)	85 (74-93)

Abbreviations: IQR, Inter-Quartile Range; Mdn, Median.

### Subtype-specific analyses

There were no differences (*P* > .05) in median HRQoL scores between MEN subtypes (MEN1, MEN2A and MEN2B) across most domains. However, a difference did emerge for Physical Functioning, χ^2^ (2) = 6.56, *P* = .038. Pairwise comparisons indicated that participants with MEN2B reported lower median scores in the Physical Functioning domain (*MdN* = 72) compared to those with MEN1 (*MdN* = 94, *P* = .011) and MEN2A (*MdN* = 97, *P* = .016). No other group differences were observed.

### Age-related trends

There were no differences (*P* > .05) in median HRQoL scores between age categories (5-7, 8-12, and 13-18 years) across most domains. However, a difference did emerge for School Functioning, χ^2^ (2) = 6.13, *P* = .047. Pairwise comparisons indicated that children aged 5-7 reported higher median scores in the School Functioning domain (*MdN* = 90) compared to those aged 8-12 (*MdN* = 70, *P* = .015) and 13-18 (*MdN* = 80, *P* = .028). No other group differences were observed.

### Comparison with siblings and gender differences

Mann–Whitney *U* tests revealed no differences (*P* > .05) between children with MEN and their siblings without MEN in median HRQoL scores across all domains. Within the group of children with MEN, no gender differences (*P* > .05) were found across all domains of the PedsQL. Among siblings, however, boys reported higher scores than girls on the Total (*P* = .019), Physical (*P* = .005), and Emotional (*P* = .039) domains.

### Parent-proxy scores vs child self-reported outcomes

Proxy outcomes corresponding to children who also completed the self-report questionnaire are presented across age categories and MEN subtypes as medians and interquartile ranges (IQRs) in Table 4. Corresponding mean and standard deviation (SD) values are provided in Table S2 (25). Proxy outcomes were matched with sixty-eight self-report outcomes. Both mothers (*N* = 58) and fathers (*N* = 10) completed the proxy questionnaire regarding their affected child(ren). Wilcoxon signed-rank tests revealed systematic differences between proxy- and self-reported median scores. Parents’ proxy reports indicated higher HRQoL scores for their children than the children's own self-reported scores across nearly all domains: Total (*z* = 3.50, *P* < .001), Physical (*z* = 2.11, *P* = .034), Social (*z* = 2.98, *P* = .003), School (*z* = 2.56, *P* = .010), and Psychosocial (*z* = 3.45, *P* < .001), except for Emotional Functioning (*z* = 1.74, *P* = .082), where no significant discrepancy was observed.

**Table 4:** Matched proxy HRQoL outcomes per age category and subtype of MEN

		Physical	Emotional	Social	School/Work	Psychosocial	Total
	N	Mdn (IQR)	Mdn (IQR)	Mdn (IQR)	Mdn (IQR)	Mdn (IQR)	Mdn (IQR)
**Total**	**68**	**97 (91-100)**	**90 (70-100)**	**100 (95-100)**	**85 (66-100)**	**91 (78-97)**	**90 (84-98)**
8-12	32	97 (91-100)	85 **(70-100)**	100 (86-100)	85 **(66-100)**	89 (77-98)	89 **(83-97)**
13-18	36	97 (91-100)	90 **(73-100)**	100 (100-100)	90 **(66-100)**	93 (78-97)	92 **(94-98)**
**MEN1**	**27**	**100 (94-100)**	**90 (75-100)**	**100 (100-100)**	**90 (75-100)**	**93 (83-100)**	**96 (88-99)**
8-12	10	100 (95-100)	95 **(68-100)**	100 (99-100)	95 **(68-100)**	97 (79-100)	97 **(86-99)**
13-18	17	100 (94-100)	90 **(75-98)**	100 (100-100)	90 **(75-100)**	93 (82-97)	95 **(87-98)**
**MEN2A**	**37**	**94 (91-100)**	**90 (70-100)**	**100 (83-100)**	**80 (65-95)**	**90 (77-96)**	**89 (79-96)**
8-12	20	94 (91-100)	83 **(70-100)**	100 (81-100)	80 **(65-90)**	88 (77-95)	88 **(78-95)**
13-18	17	97 (91-100)	100 **(70-100)**	100 (90-100)	80 **(63-100)**	90 (78-98)	90 **(79-99)**
**MEN2B**	**4**	**80 (68-89)**	**80 (69-91)**	**90 (83-98)**	**85 (70-89)**	**81 (79-92)**	**83 (76-87)**
8-12	2	88 (−)	73 **(−)**	85 (−)	85 **(−)**	81 (−)	83 **(−)**
13-18	2	70 (−)	88 **(−)**	95 (−)	78 **(−)**	87 (−)	81 **(−)**

Abbreviations: IQR, Inter-Quartile Range; Mdn, Median.

### Lowest HRQoL group

No differences (*P* > .05) between *at risk* and *normal* scorers were observed in exploratory analyses for sex, MEN subtype, age category, history of surgeries, mutation risk category, or family history of MEN-related deaths. However, Chi-square analyses indicated that a higher proportion of children with clinical manifestations of MEN (*N* = 16) fell within the *at-risk* group for Physical Functioning (χ^2^(1, *N* = 76) = 7.38, *P* = .007, Cramer's V = .31), Social Functioning (χ^2^(1, *N* = 76) = 7.18, *P* = .007, Cramer's V = .31), and School Functioning (χ^2^(1, *N* = 76) = 4.91, *P* = .027, Cramer's V = .25) compared to children with no disease manifestations. Notably, all observed clinical manifestations, hyperparathyroidism, pancreatic neuroendocrine tumors, pituitary adenomas, medullary thyroid carcinoma, and other MEN-related manifestations, occurred within the MEN1 and MEN2B subgroups, whereas none were reported among children with MEN2A (Table 2). Although children with any clinical manifestation were more likely to fall within the *at-risk* range for Physical, Social, and School Functioning, the small numbers within each manifestation type did not allow to distinguish whether specific manifestations were more strongly associated with HRQoL impairments.

## Discussion

Despite the fact that children diagnosed with MEN syndrome are at risk to develop endocrine disease manifestations and require lifelong screening, children with MEN syndrome report an overall HRQoL comparable to siblings and age-matched Dutch norms. Only children diagnosed with MEN2B reported reduced physical functioning. Although younger children with MEN syndrome reported better school functioning than older children, no gender differences were observed within the MEN group. In contrast, among siblings, girls reported lower HRQoL across multiple domains compared to boys. Discrepancies between child- and parent-reported HRQoL highlight the importance of incorporating both perspectives in clinical practice. In the subgroup of children with MEN who already presented with clinical manifestations, physical, social, and school functioning were negatively affected. This pattern suggests that, once disease symptoms emerge, early identification of psychosocial difficulties and timely, targeted support may be crucial to maintaining daily functioning and mitigating the impact of illness.

Rather than demographic or genetic variables, it was found that only symptomatic disease was associated with lower HRQoL across several domains. These findings suggest that HRQoL may be more closely related to clinical expression than to genotype or background characteristics. The lower scores on Physical Functioning amongst children with MEN2B, together with the clustering of clinical manifestations within the MEN1 and MEN2B subgroups, align with known differences in disease severity and onset across subtypes (26). Younger children showed higher scores on School Functioning, which is consistent with previous studies in general populations, indicating that younger children generally report better HRQoL than (pre)adolescents (27, 28). It should be noted, however, that outcomes for children aged 5-7 were based exclusively on proxy reports, and awareness or understanding of their diagnosis was not assessed, which complicates direct comparison.

Comparisons between child self-reports and parent proxy-reports demonstrated systematic discrepancies, with parents generally reporting higher HRQoL scores than their children. This contrasts with previous research in other populations, which commonly shows that parents tend to rate their child's functioning lower than the child's own reports (29, 30). Higher parent-reported HRQoL scores have primarily been observed in non-clinical populations instead of children with health conditions (31). Overall, these findings could reflect optimism or protective coping mechanisms among parents in families affected by MEN.

Sibling comparisons further contextualize these outcomes. Siblings reported HRQoL scores comparable to normative data, suggesting that, unlike siblings of children with chronic illnesses such as epilepsy, diabetes, or cerebral palsy, siblings of children with MEN seem to have a relatively preserved HRQoL and resilience despite the presence of MEN in their family (32). This may be explained by the development of adaptive family coping strategies. Further research on coping in children with MEN and their siblings could provide important context for these findings. Within the group of siblings, boys reported significantly higher HRQoL than girls across most domains, consistent with broader pediatric literature showing lower HRQoL in girls, particularly in psychosocial domains (28, 33). This is usually explained by higher rates of internalizing symptoms in girls, such as anxiety and mood-related symptoms, which contribute to lower HRQoL. In this context, it is more surprising that these differences were not found in the group of children with MEN. This may reflect that living with a chronic hereditary condition activates adaptive coping processes, which could mitigate typical gender-related variations in HRQoL. Additionally, children with MEN grow up in families familiar with the disease and under close medical follow-up, and therefore may experience greater perceived familial and clinical support, potentially buffering gender disparities that remain visible among siblings without MEN.

Several limitations should be considered; the PedsQL is a generic questionnaire and not specific to hereditary tumor syndromes. However, it is a well-validated and widely used instrument that allows comparison with Dutch population norms. The cross-sectional nature of the data prevents causal inference, but it provides an essential first national overview of HRQoL in this population. As the number of individual clinical manifestations was small, it could not be determined whether specific manifestations affected HRQoL to a greater or lesser extent. Larger studies are needed to clarify whether certain manifestations carry a greater psychosocial burden. Finally, the online data collection might have excluded families with lower digital literacy, but participation rates (±73%) were high and covered all known pediatric MEN cases in the Netherlands, supporting representativeness. Overall, the study's comprehensive recruitment, inclusion of sibling and proxy comparisons, and analysis of multiple explanatory factors offer robust and new insights into HRQoL of children with MEN.

Together, these findings are reassuring and highlight the overall resilience of children with MEN. However, regular psychosocial screening might be particularly warranted for those with clinical manifestations and children with MEN1 or MEN2B syndrome. As clinical manifestations were observed only in MEN1 and MEN2B, these subgroups may benefit most from closer psychosocial monitoring and tailored support. The observed discrepancies between child- and parent-reported HRQoL underscore the importance of integrating both perspectives in clinical practice to obtain a comprehensive understanding of the child's well-being. Future research could also explore what children at a young age actually understand about MEN and its implications, and how this awareness (or lack thereof) relates to their perceived quality of life. Additionally, further studies may focus on coping mechanisms and family dynamics in children with MEN and their siblings to further contextualize these findings. Longitudinal studies could help elucidate HRQoL trajectories and examine the effects of disease manifestations, treatment history, and comorbidities. Overall, these results show that, despite the diagnosis of a familial tumor predisposition syndrome, HRQoL may not be diminished, which is reassuring and important information in the counseling of these families. For patients with clinical manifestations, a holistic approach is needed that integrates medical and psychosocial support, enabling early identification and intervention in at-risk domains and strengthening family-centered coping and overall quality of life.

## Conclusion

In conclusion, this nationwide study provides new insights into HRQoL in children with MEN1, MEN2A, and MEN2B. HRQoL scores were generally comparable to those of siblings and healthy, age-matched Dutch norms, with the exception of reduced physical functioning in children with MEN2B. In addition, the presence of clinical manifestations was associated with lower HRQoL in physical, social, and school functioning domains. As clinical manifestations occurred only in MEN1 and MEN2B, these subgroups may benefit from closer psychosocial monitoring and targeted support. The discrepancies between child- and parent-reported HRQoL further underscore the importance of integrating both perspectives into clinical practice to ensure a comprehensive understanding of the child's well-being.

## Data Availability

Some or all datasets generated during and/or analyzed during the current study are not publicly available but are available from the corresponding author on reasonable request.

## References

[1] Romei C, Pardi E, Cetani F, Elisei R. Genetic and clinical features of multiple endocrine neoplasia types 1 and 2. J Oncol. 2012;2012:1‐15. 10.1155/2012/705036 PMC3503399 23209466

[2] van Leeuwaarde RS, de Laat JM, Pieterman CRC, Dreijerink K, Vriens MR, Valk GD. The future: medical advances in MEN1 therapeutic approaches and management strategies. Endocr Relat Cancer. 2017;24(10):T179‐T193. 28768698 10.1530/ERC-17-0225

[3] Pieterman CRC, Vriens MR, Dreijerink KMA, van der Luijt RB, Valk GD. Care for patients with multiple endocrine neoplasia type 1: the current evidence base. Fam Cancer. 2011;10(1):157‐171. 21061174 10.1007/s10689-010-9398-6

[4] Kamilaris CDC, Stratakis CA. Multiple endocrine neoplasia type 1 (MEN1): an update and the significance of early genetic and clinical diagnosis. Front Endocrinol (Lausanne). 2019;10:339. 31263451 10.3389/fendo.2019.00339 PMC6584804

[5] de Laat JM, van der Luijt RB, Pieterman CRC, et al MEN1 redefined, a clinical comparison of mutation-positive and mutation-negative patients. BMC Med. 2016;14(1):182. 27842554 10.1186/s12916-016-0708-1 PMC5109674

[6] Pieterman CRC, van Leeuwaarde RS, van den Broek MFM, van Nesselrooij BPM, Valk GD. Multiple endocrine neoplasia type 1. In: Feingold KR, Adler RA, Ahmed SF, et al., eds. *Endotext*. MDText.com, Inc.; December 22, 2021.

[7] Jacob M, Rowland D, Lekarev O, Ergun-Longmire B. Multiple endocrine neoplasia in childhood: an update on diagnosis, screening, management and treatment. Endocrines. 2022;3(1):76‐91.

[8] Paun D, Tilici D, Paun S, Mirica A. Prospective genetic screening in multiple endocrine neoplasia syndromes. Children. 2024;11(8):1012. 39201946 10.3390/children11081012 PMC11352621

[9] Bakhuizen JJ, Hopman SMJ, Bosscha MI, et al Assessment of cancer predisposition syndromes in a national cohort of children with a neoplasm. JAMA Netw Open. 2023;6(2):e2254157. 36735256 10.1001/jamanetworkopen.2022.54157 PMC9898819

[10] Brandi ML, Pieterman CRC, English KA, et al Multiple endocrine neoplasia type 1 (MEN1): recommendations and guidelines for best practice. Lancet Diabetes Endocrinol. 2025;13(8):699‐721. 40523372 10.1016/S2213-8587(25)00119-6

[11] Vetsch J, Wakefield CE, Warby M, et al Cancer-related genetic testing and personalized medicine for adolescents: a narrative review of impact and understanding. J Adolesc Young Adult Oncol. 2018;7(3):259‐262. 29336661 10.1089/jayao.2017.0102

[12] van Engelen K, Barrera M, Wasserman JD, et al Tumor surveillance for children and adolescents with cancer predisposition syndromes: the psychosocial impact reported by adolescents and caregivers. Pediatr Blood Cancer. 2021;68(8):e29021. 33788392 10.1002/pbc.29021

[13] Waldmann J, Fendrich V, Habbe N, et al Screening of patients with multiple endocrine neoplasia type 1 (MEN-1): a critical analysis of its value. World J Surg. 2009;33(6):1208‐1218. 19350320 10.1007/s00268-009-9983-8

[14] Gopie JP, Vasen HFA, Tibben A. Surveillance for hereditary cancer: does the benefit outweigh the psychological burden? A systematic review. Crit Rev Oncol Hematol. 2012;83(3):329‐340. 22366115 10.1016/j.critrevonc.2012.01.004

[15] van Leeuwaarde RS, Pieterman CRC, May AM, et al Health-related quality of life in patients with multiple endocrine neoplasia type 1. Neuroendocrinology. 2020;111(3):288‐296. 32365349 10.1159/000508374

[16] van Leeuwaarde RS, Pieterman CRC, Bleiker EMA, et al High fear of disease occurrence is associated with low quality of life in patients with multiple endocrine neoplasia type 1: results from the Dutch MEN1 study group. J Clin Endocrinol Metab. 2018;103(6):2354‐2361. 29618015 10.1210/jc.2018-00259

[17] Berglund G, Lidén A, Hansson MG, Oberg K, Sjöden PO, Nordin K. Quality of life in patients with multiple endocrine neoplasia type 1 (MEN 1). Fam Cancer. 2003;2(1):27‐33. 14574164 10.1023/a:1023252107120

[18] Zwerus D, Strobbe F, Verrijn Stuart AA, et al Psychosocial outcomes in patients with endocrine tumor syndromes: a systematic review. Pediatr Blood Cancer. 2026;73(3):e70037. 41491677 10.1002/1545-5017.70037

[19] Lyon ME, Wiener L. Special issue: psychosocial considerations for children and adolescents living with a rare disease. Children. 2022;9(7):1099. 35884083 10.3390/children9071099 PMC9322344

[20] Witt S, Schuett K, Wiegand-Grefe S, Boettcher J, Quitmann J. Living with a rare disease—experiences and needs in pediatric patients and their parents. Orphanet J Rare Dis. 2023;18(1):242. 37568186 10.1186/s13023-023-02837-9 PMC10422846

[21] Varni JW, Seid M, Kurtin PS. PedsQL^TM^ 4.0: reliability and validity of the pediatric quality of life Inventory^TM^ version 4.0 generic core scales in healthy and patient populations. Med Care. 2001;39(8):800‐812. 11468499 10.1097/00005650-200108000-00006

[22] Engelen V, Haentjens MM, Detmar SB, Koopman HM, Grootenhuis MA. Health related quality of life of Dutch children: psychometric properties of the PedsQL in The Netherlands. BMC Pediatr. 2009;9(1):68. 19887000 10.1186/1471-2431-9-68 PMC2776579

[23] Dunn OJ . Multiple comparisons using rank sums. Technometrics. 1964;6(3):241‐252.

[24] Wells SA, Asa SL, Dralle H, et al Revised American thyroid association guidelines for the management of medullary thyroid carcinoma. Thyroid. 2015;25(6):567‐610. 25810047 10.1089/thy.2014.0335 PMC4490627

[25] Zwerus D, Verrijn Stuart AA, Van Santen HM, et al 2026. Supplementary data: health-related quality of life in children with multiple endocrine neoplasia (MEN) and their siblings [Internet]. [cited 2026 Feb 16]. Available from Open Science Framework: 10.17605/OSF.IO/24ZMU PMC13368371 41779164

[26] Hu X, Guan J, Wang Y, et al A narrative review of multiple endocrine neoplasia syndromes: genetics, clinical features, imaging findings, and diagnosis. Ann Transl Med. 2021;9(11):944‐944. 34350259 10.21037/atm-21-1165 PMC8263874

[27] Michel G, Bisegger C, Fuhr DC, Abel T. Age and gender differences in health-related quality of life of children and adolescents in Europe: a multilevel analysis. Qual Life Res. 2009;18(9):1147‐1157. 19774493 10.1007/s11136-009-9538-3

[28] Svedberg P, Eriksson M, Boman E. Associations between scores of psychosomatic health symptoms and health-related quality of life in children and adolescents. Health Qual Life Outcomes. 2013;11(1):176. 24148880 10.1186/1477-7525-11-176 PMC3831247

[29] Jeanbert E, Baumann C, Todorović A, Tarquinio C, Rousseau H, Bourion-Bédès S. Factors associated with discrepancy of child-adolescent/parent reported quality of life in the era of COVID-19. Int J Environ Res Public Health. 2022;19(21):14359. 36361238 10.3390/ijerph192114359 PMC9654617

[30] Eiser C, Varni JW. Health-related quality of life and symptom reporting: similarities and differences between children and their parents. Eur J Pediatr. 2013;172(10):1299‐1304. 23715654 10.1007/s00431-013-2049-9

[31] Upton P, Lawford J, Eiser C. Parent–child agreement across child health-related quality of life instruments: a review of the literature. Qual Life Res. 2008;17(6):895‐913. 18521721 10.1007/s11136-008-9350-5

[32] Dinleyici M, Carman KB, Özdemir C, et al Quality-of-life evaluation of healthy siblings of children with chronic illness. Balkan Med J. 2019;37(1)34‐42. 31647208 10.4274/balkanmedj.galenos.2019.2019.7.142 PMC6934013

[33] Dłużniak-Gołaska K, Szostak-Węgierek D, Panczyk M, Szypowska A, Sińska B. May gender influence the quality of life in children and adolescents with type 1 diabetes? Patient Prefer Adherence. 2019;13:1589‐1597. 31571841 10.2147/PPA.S206969 PMC6759230

[34] Falchetti A, Marini F, Luzi E, et al Multiple endocrine neoplasia type 1 (MEN1): not only inherited endocrine tumors. Genet Med. 2009;11(12):825‐835. 19904212 10.1097/GIM.0b013e3181be5c97

[35] Krampitz GW, Norton JA. RET gene mutations (genotype and phenotype) of multiple endocrine neoplasia type 2 and familial medullary thyroid carcinoma. Cancer. 2014;120(13):1920‐1931. 24699901 10.1002/cncr.28661

[36] Li SY, Ding YQ, Si YL, Ye MJ, Xu CM, Qi XP. 5P strategies for management of multiple endocrine neoplasia type 2: a paradigm of precision medicine. Front Endocrinol (Lausanne). 2020;11:543246. 33071967 10.3389/fendo.2020.543246 PMC7531599

[37] Schepers SA, van Oers HA, Maurice-Stam H, et al Health related quality of life in Dutch infants, toddlers, and young children. Health Qual Life Outcomes. 2017;15(1):81. 28438198 10.1186/s12955-017-0654-4 PMC5404665

[38] van Muilekom MM, Luijten MAJ, van Oers HA, et al Paediatric patients report lower health-related quality of life in daily clinical practice compared to new normative PedsQL ^TM^ data. Acta Paediatr. 2021;110(7):2267‐2279. 33838052 10.1111/apa.15872 PMC8360011

